# Students’ Medical Dictionary

**Published:** 1897-10

**Authors:** 


Students' Medical Dictionary—By George M. Gould, A. M., M. D., Author of "An Illustrated Dictionary of Medicine, Biology and Allied Sciences," etc. *Tenth Edition Rewritten and Enlarged.* Philadelphia: P. Blakiston, Son & Co., 1896. Small 8vo. Half-dark Leather, $3.25 net; Half Morocco. Thumb index. $4.00 net.

This edition is much superior to those which have preceded it. Many new words have been added and the book enlarged and otherwise improved. Besides containing most of the medical words and terms, it contains elaborate tables of the bacilli, micorcocci, ptomaines, leucomains, etc.; also of the arteries, muscles, nerves; of weights and measures, etc. The author has adopted the new orthography, leaving off as far as possible all silent letters in a word. The system of pronunciation adopted is a very simple one, and easily understood. The definitions are brief and clear, making them easy for the student to understand. This dictionary is intended for the medical student and it will serve well the purpose for which it is designed. H.

